# Food and water restriction lead to differential learning behaviors in a head-fixed two-choice visual discrimination task for mice

**DOI:** 10.1371/journal.pone.0204066

**Published:** 2018-09-13

**Authors:** Pieter M. Goltstein, Sandra Reinert, Annet Glas, Tobias Bonhoeffer, Mark Hübener

**Affiliations:** 1 Max Planck Institute of Neurobiology, Martinsried, Germany; 2 Graduate School of Systemic Neurosciences, Martinsried, Germany; Tokai University, JAPAN

## Abstract

Head-fixed behavioral tasks can provide important insights into cognitive processes in rodents. Despite the widespread use of this experimental approach, there is only limited knowledge of how differences in task parameters, such as motivational incentives, affect overall task performance. Here, we provide a detailed methodological description of the setup and procedures for training mice efficiently on a two-choice lick left/lick right visual discrimination task. We characterize the effects of two distinct restriction regimens, i.e. food and water restriction, on animal wellbeing, activity patterns, task acquisition, and performance. While we observed reduced behavioral activity during the period of food and water restriction, the average animal discomfort scores remained in the ‘sub-threshold’ and ‘mild’ categories throughout the experiment, irrespective of the restriction regimen. We found that the type of restriction significantly influenced specific aspects of task acquisition and engagement, i.e. the number of sessions until the learning criterion was reached and the number of trials performed per session, but it did not affect maximum learning curve performance. These results indicate that the choice of restriction paradigm does not strongly affect animal wellbeing, but it can have a significant effect on how mice perform in a task.

## Introduction

Rodents, in particular rats and mice, have long been used in behavioral studies exploring the mechanisms underlying learning and memory [1,2]. Such experiments are particularly valuable when combined with simultaneous recordings from neurons involved in the task. Traditionally, this is done with extracellular recordings of single- or multi-unit activity, a technique that can easily be adopted to freely moving animals [3]. In some instances, however, it is desirable to carry out behavioral experiments in movement-restricted animals. Head-fixation in particular is indispensable under certain conditions, e.g. when precise control over sensory inputs is needed, or when the employed recording technique is sensitive to brain motion, like patch clamp recordings [4] and two-photon microscopy [5].

Head-fixed operant conditioning is now commonly used to train mice in diverse sensory detection- and discrimination tasks, as well as in virtual navigation experiments. Such tasks can be performed using various operant-stimulus modalities, e.g. visual [6], auditory [7], olfactory [8] or tactile [9]. The most widely used paradigm is the Go/No-Go task, in which the animal makes a choice by either performing or withholding from a certain behavior, such as a lick on a lick spout [9], a lever press [6] or running [10]. An important factor in behavioral training, especially with parallel neuronal recordings, is the ability to differentiate between the actual choice of a mouse and the mere level of motivation to participate in a task. Go/No-Go task designs lack the ability to precisely differentiate between an active No-Go (active withholding) and a passive No-Go, reflecting loss of motivation. Two-choice designs are therefore often more appropriate as they better allow discriminating between active choices of a mouse, e.g. licks left or right [9] or steering wheel movements to the left or right [11], and its task engagement (finished versus missed trials). Head-fixed paradigms also vary in the dimensionality of body movement that is permitted and measured. While some virtual reality approaches allow more degrees of freedom [12–14], it is common to restrict running to one dimension [10,15] or restrict body movement entirely by placing the animal in a narrow tube [9,16]. Beyond these, many more detailed parameters, e.g. setup design, training protocol, trial sequence and stimulus presentation can be adjusted to suit the specific experimental need.

The effect of such parameter choices on the outcome of a behavioral experiment is often not systematically explored and only occasionally reported in the literature. One such parameter is the choice of (naturalistic) motivational incentive. This can be appetitive (e.g. reward) or aversive (e.g. fear) and is commonly administered by delivery of food or water [9] or by delivery of mild shocks, respectively [17]. Animal behavior can also be motivated using targeted, optogenetic activation of dopaminergic circuits [11] or by circuits driving hunger or thirst [18]. Still, head-fixed learning paradigms mostly use food and water restriction, in part because it does not require additional optical equipment. While food and water restriction regimens are sometimes perceived as interchangeable, these two methods engage the animal’s physiology differently [19,20], and hunger and thirst recruit different neuronal circuits [18,21,22]. Therefore, similar levels of food and water restriction, as usually measured by the animal’s relative reduction in body weight, might affect task performance, task motivation and also animal welfare in a different manner [23].

This study provides a detailed description of the setup design and procedures to efficiently train mice using either food or water restriction on an appetitive operant visual discrimination task. We explicitly monitor animal welfare using measurements of body weight and a standardized scoring routine, as well as continuously recorded physical activity patterns from the home cage [24]. We demonstrate the sensitivity and reliability of our conditioning method by addressing how the choice for food or water restriction affects performance in head-fixed operant conditioning.

## Methods

### Animals

All procedures were performed in accordance with the institutional guidelines of the Max Planck Society and the local government (protocol number 55.2-1-54-2531-213-2015, approved by the Beratende Ethikkommission nach § 15 Tierschutzgesetz, Regierung von Oberbayern). Twelve male C57BL/6J mice (postnatal day 34) were individually housed in standard individually ventilated cages (IVC; Tecniplast GM500) and placed in a Digital Ventilated Rack (DVC, Tecniplast). Each cage was equipped with a dedicated electronic board (DVC board) composed of 12 electromagnetic field generating electrodes evenly positioned in a 4 by 3 grid underneath the entire cage floor area. Sensors measured activity at each electrode separately (4 Hz sampling frequency) and stored the data on a computer. Disturbances in the strength of the local electromagnetic field were used as proxy for a mouse’s behavioral activity in the home cage (see Data analysis). All mice were kept on an inverted 12-h light, 12-h dark cycle with lights on at 22:00. Ambient temperature (21.0 ± 0.7 ^o^C) and humidity (63 ± 2%) were kept constant. Water and standard chow (Altromin Spezialfutter GmbH, #1310) were provided to the mice *ad libitum* prior to behavioral experiments. Starting seven days before surgery, mice were handled and weighed daily by the same experimenters (two female, two male) that later also carried out behavioral training. After completion of behavioral procedures, mice were euthanized using CO_2_ asphyxiation.

### Surgical procedures

**Fig 1:**
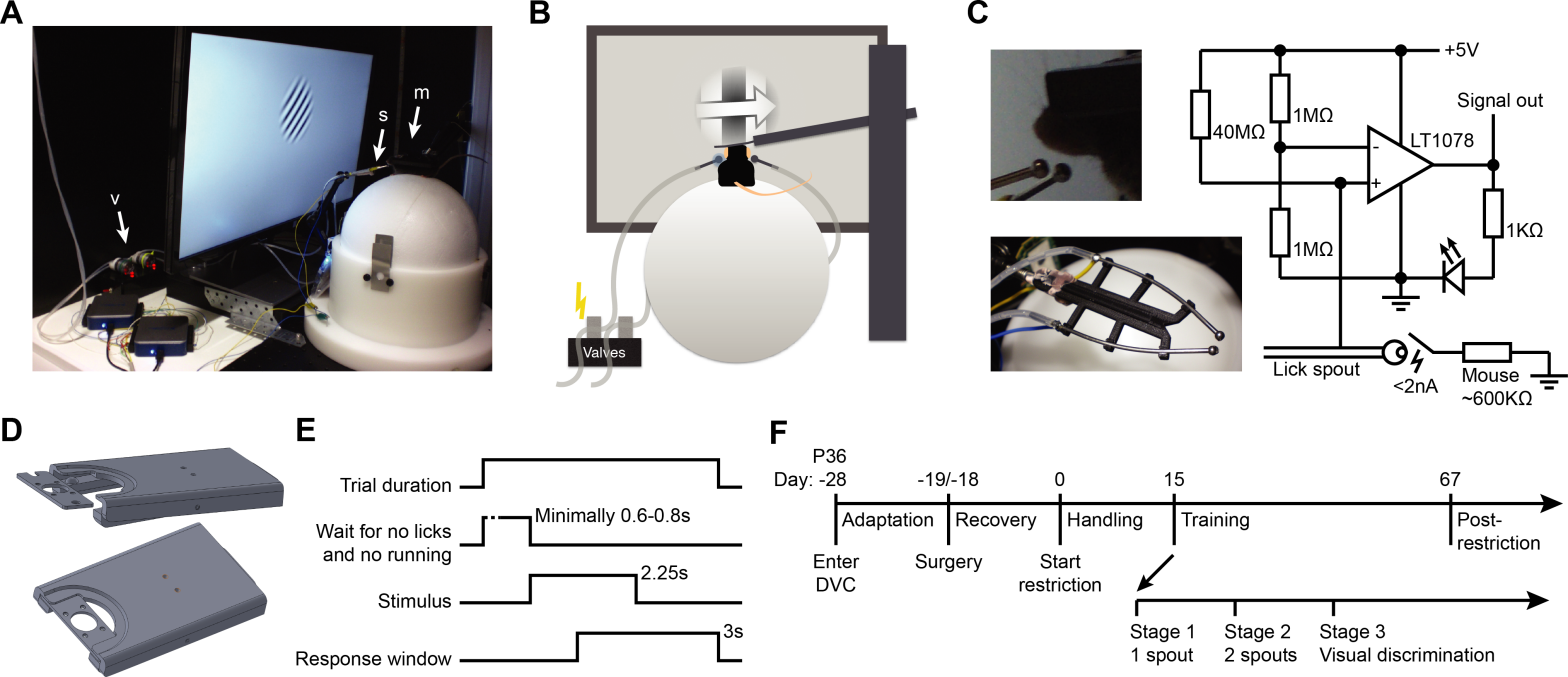
Behavioral apparatus and training protocol. A. Setup used for head-fixed visual conditioning. Arrow ‘m’ points to a head-fixed mouse, resting on a Styrofoam ball, in front of a centrally positioned monitor and the two lick spouts (arrow ‘s’). Arrow ‘v’ indicates the pinch valves for reward delivery. B. Schematic of the behavioral setup as seen from behind. C. Lick detection. Top left: Position of dual lick spouts in front of the mouse. Bottom left: Photo of fully assembled 3D printed lick spout holder. Right: Electrical circuit for contact/lick detection on a single lick spout. D. 3D renderings of head-bar and head-bar holder. E. Temporal sequence of within-trial phases. Reward is delivered immediately upon the first (correct) lick in the response window. F. Overall experimental timeline depicting main experimental and training stages.

Mice were anesthetized with a mixture of fentanyl, midazolam and meditomidine in saline (0.05mg/kg, 5 mg/kg and 0.5 mg/kg respectively, injected i.p.) and sufficient depth of anesthesia was confirmed by absence of the pedal reflex. Eyes were covered with a thin layer of ophthalmic ointment (IsoptoMax). Lidocaine (0.2mg/ml) was applied onto the scalp for topical anesthesia and carprofen in saline (5mg/kg, injected s.c.) was administered for analgesia. The skull was exposed, dried and scraped with a scalpel to facilitate attachment of the head plate. The custom-designed head plate (Fig 1D; S1 File) was fixed in position, over the left parietal bone, using cyanoacrylate glue and subsequently secured with dental acrylic (Paladur). After surgery, mice were injected with a mixture of the antagonists naloxone, flumazenil and atipamezole in saline (1.2 mg/kg, 0.5 mg/kg and 2.5mg/kg respectively, injected s.c.) and left to recover under a heat lamp. For post-operative analgesia, mice received carprofen (5mg/kg, injected s.c.) for three subsequent days.

### Food and water restriction

Mice were randomly assigned to either the food-restricted or the water-restricted group. The period of restricted access to food or water was started 18–19 days after surgery. Animals were transferred to novel cages immediately before food or water restriction started.

At the start of water access restriction, mice were initially provided with 50% of the average *ad libitum* water intake per day (50% was on average 1.57 ml in our facility). The water ration was provided in the home cage using the nozzle of a standard water bottle that was closed off at the back using red tape. From the fourth day onwards, the water ration was first offered in a hand-held syringe during handling, with any remaining volume supplied in the home cage. In parallel, when a mouse reached the target weight of 85% of the initially measured *ad libitum* weight (reference weight), the daily volume of water supplement was individually adjusted in order to maintain the target weight. As precaution, a minimum daily ration was set to 25% of the *ad libitum* intake. However, the daily supplemented amount was only rarely as low as 25% of *ad libitum* intake. Water-restricted animals had *ad libitum* access to food throughout the experiment.

Food access was restricted according to the following procedure. On the first day, mice received the minimum ration size of 2.0 g standard chow (3.279 kcal/g) in their home cage. Subsequently, the daily ration was adjusted per mouse in order to keep its weight at 85% of the reference weight, while staying above the minimum ration weight of 2.0 g. From day four untill day seven, mice were fed unflavored soymilk (Alpro) from a handheld syringe during handling. However, we noted that mice were not particularly motivated to drink regular soymilk (average consumed volume per mouse; day 5: 0.58 ml; day 6: 0.63 ml; day 7: 0.51 ml). Thus, on days eight and nine we offered sweetened soymilk (Alpro), which did not increase the consumed volume (average consumed volume per mouse; day 8: 0.51 ml; day 9: 0.38 ml). Finally, from the tenth day until the end of the experiment we used infant formula soymilk (SMA Wysoy)[10]. The infant formula soymilk was prepared by adding lukewarm water to a falcon tube containing 10–12 ml of soymilk powder until the total volume of the suspension reached 30–34 ml. We immediately noticed a difference in consumption behavior when providing mice with infant formula soymilk (average consumed volume per mouse; day 7: 0.73 ml; day 8: 1.29 ml; day 9: 0.99 ml; day 10: 1.73 ml; day 11: 1.71 ml). The daily food ration of each mouse was reduced by an amount that matched the caloric content of the consumed volume of soymilk (0.67 kcal/ml). Food-restricted animals had *ad libitum* access to water during the entire experiment.

### Animal welfare assessment

Daily welfare assessment involved scoring mice on five different aspects of wellbeing using individual scoresheets [9]. Scores on ‘Activity and behavior’ ranked an animal’s behavior in the home cage from normal, active (0), via reduced activity (1), only moves when touched (2) to lethargy (3). ‘Look/posture’ indicated the condition of the fur and the posture of the mouse, ranging from normal (0) to arched back and very shaggy fur (3). ‘Urine/feces’ was scored as indication of eating, drinking and associated physiological processes, ranging from normal (0), via reduced amounts (1) to none (2). ‘Body condition’ indicated the shape and outline of the mouse’s body and spine according to Ullman-Culleré & Foltz [25], ranging from normal (0), via underweight (1) to emaciated (2). ‘Signs of dehydration’ were assessed using skin turgor, ranging from none (0), via light (1), moderate (2), to strong (3). Finally, the cumulative score across all aspects was used to judge the overall wellbeing of the animal into four discomfort categories named according to the European Union based legislation. A discomfort score of zero was interpreted as ‘sub-threshold discomfort’, one as ‘mild discomfort’, between two and four as ‘moderate discomfort’ and higher than four as ‘severe discomfort’.

### Apparatus for visual discrimination learning

Visual discrimination learning was carried out in custom-built setups that were placed in 75 x 75 x 75 cm boxes, providing a semi-enclosed environment (Fig 1A and 1B). The apparatus consisted of a head-plate holder, a spherical treadmill, a computer monitor, two lick spouts (16 Gauge, 3mm tip-diameter reusable feeding needles, Fine Science Tools) with lick detectors, tubes and valves to supply liquid reward. The treadmill was made of an airflow-supported Styrofoam ball [26] and restricted to forward and backward motion by a pin pushed into the side of the ball. An optical sensor, extracted from a computer mouse (G502, Logitech), tracked rotation of the ball using a custom-written LabVIEW (National Instruments) program. The mouse was head-fixed on the ball using a surgically implanted aluminum head plate, clamped into a custom-designed holder (S1 File). The head plate holder employed a (simplified) system of kinematic mounts to ensure reproducible positioning of the animal’s head within the apparatus (Fig 1D; S1 File; [27]). Visual stimuli were presented on a gamma corrected computer monitor (Dell P2414H; resolution: 1920 by 1080 pixels; width: 52.8 cm; height 29.6 cm; maximum luminance: 182.3 Cd/m^2^). The monitor was positioned in front of the mouse at a distance of 18 cm and centered at 0 degrees azimuth and elevation. The box was illuminated by red LEDs (630 nm), and a webcam (Logitech F100) was used to observe the mouse and setup within the enclosed space.

The two steel lick spouts were mounted on a custom 3D-printed holder that allowed fine adjustment of the space between the lick spout nozzles (S2 File). The lick spouts were positioned in front of the animals’ mouth using a movable arm (Fig 1C, left panels). Care was taken to place the lick spout well within the reach of the tongue, which is especially important in the first pre-training sessions. Precise central positioning of the lick spouts with respect to the animal’s mouth was critical; asymmetrical placement sometimes biased mice to make more licks on the closer spout. Each lick spout was connected to a custom-made lick detection circuit based on Weijnen [28] and Slotnick [29]. The circuit registered a voltage drop on the non-inverting high impedance input of an operational amplifier (LT1079CN; Linear Technologies) when the mouse short-circuited the input by licking on the spout (Fig 1C, right panel). The inverting input was connected to a voltage divider such that an individual lick triggered a strong discrete voltage drop in the amplifier output. The non-inverting and inverting inputs of the circuit could be switched in order for the circuit to report licks by voltage peaks. However, the described arrangement allows detecting whether the circuit is switched on from the baseline circuit output voltage.

Liquid reward was supplied through the lick spout by gravitational flow, operated using full opening pinch valves (NResearch Inc.). Valves were individually calibrated to supply drops of approximately 8 μl, which required valve-open durations of roughly 50 ms for water and approximately 75 ms for soymilk. Tubing was pressure-flushed with distilled water after each behavioral training session to prevent clogging. Signals from the lick detectors, the optical speed sensor and other triggers were recorded with two USB multifunction input/output devices (USB6001, National Instruments). The first device was used for closed loop control of the setup using a custom-written behavioral-training program (Matlab, Mathworks). The second device passively recorded all sensor signals at 500 Hz using a custom-written data-acquisition program (LabVIEW, National Instruments), which allowed for more precise offline analysis of behavioral parameters (see Data analysis).

### Habituation and pre-training for head-fixed two-choice operant conditioning

Behavioral procedures were carried out six times per week between 14:00 and 18:00. In a two-week period prior to head-fixed operant training, mice were habituated to the experimental procedures. Each habituation session lasted 10 to 15 minutes during which the mouse was (1) held in the experimenter's gloved hands, (2) placed on the surface of a Styrofoam ball, (3) fed water or soymilk through a syringe and (4) accustomed to brief head fixation by holding the head plate manually for a few seconds. In this specific experiment animals were habituated for a period of two weeks because we tested different variants of soymilk (see above). However, mice typically accustom to these procedures in three to four days.

In order to shape animals for the head-fixed visual two-alternative choice task, we implemented two stages of head-fixed pre-training. The first stage familiarized animals with the association between timed licks and liquid reward from a single lick spout. To this end, animals were exposed to the trial sequence (Fig 1E), but in absence of visual stimulus presentation. Each trial started with an inter-trial interval of 2.0 s, after which the mouse was required to withhold licking and cease running (velocity below 1 cm/s) for a duration of at least 0.6 s to 0.8 s (varied per trial in order to prevent mice from learning a fixed timing sequence). When this requirement was met, the trial proceeded with the visual stimulus period. In pre-training stage 1 and 2 no actual visual stimulus was presented in this period, the screen remained blank. After 1.0 s from the onset of the visual stimulus period, the mouse could make a lick on the fluid spout in order to receive a single drop (approximately 8 μl) of water or soymilk. This period, during which a lick on the spout initiated reward delivery (named ‘response window’), lasted initially 15.0 s and was gradually reduced to 5.0 s in subsequent pre-training sessions. At the start of the training sessions, a few drops were given by manual activation of the valves in order to motivate the mouse to lick for reward and to adjust the lick spout’s positioning relative to the mouth and tongue. Mice proceeded to the second pre-training stage when they performed about 50 rewarded trials per training session on two consecutive days.

In pre-training stage 2 the trial sequence remained the same, except that now two lick spouts were positioned in front of the animal. On each trial, only a single lick spout was selected as active, and only a lick on this spout, during the response window, triggered reward delivery. Licks on the non-active spout were recorded but did not abort the remaining period of the trial/response window. The distance between the left and right lick spout was initially set to 1.0 to 1.5 mm. Later-on in pre-training stage 2, the inter-spout distance was increased to approximately 3.0–4.0 mm, the inter-trial interval was increased to 4.0 s and the response window duration was reduced to 4.0 s. Mice proceeded to the visual discrimination task when, in pre-training stage 2, animals performed a minimum of 50 trials per session and consumed drops without a strong preference for one of the two lick spouts.

### Side bias correction strategy

Mice tend to develop a strategy of responding with a majority of the licks on only one of the two lick spouts (i.e. they showed a ‘side-bias’), which we aimed to prevent using the following strategy. On each trial, we drew a random number *r* between -1 and +1. If this number was above an adjustable threshold *t*_*b*_ (bias-threshold), the next trial would give reward on the left spout, otherwise it would give reward on the right lick spout (Eq 1).

$$Next\;trial,side=(r>t_{b}\to Left)\wedge (r\leq t_{b}\to Right)$$ Eq 1

The value of the threshold *t*_*b*_ was calculated using the outcome of the last 20 non-missed trials where *n*_*correct left*_ and *n*_*correct right*_ were the total number of trials in which the first lick in the response window was on the correct spout, and *n*_*total left*_ and *n*_*total right*_ were the total number of presented ‘left trials’ and ‘right trials’ within the 20-trial period (Eq 2).

$$t_{b}=\min\;\left\{m,\max\;\left\{-m,(\left(\frac{n_{correct\;left}}{n_{total\;left}})-(\frac{c_{correct\;right}}{n_{total\;right}}\right))\right\}\right\}$$ Eq 2

The value of *m* instated a minimum probability for either stimulus to be selected by bounding the value of *t*_*b*_ to the range -*m* to +*m*. Thus, when a mouse would only lick on the left lick spout, the value of *t*_*b*_ would approximate *m*, reducing the chance that the next trial would be a ‘left trial’ to minimally 0.5−m2 and increasing the chance that the next trial would be a ‘right trial’ to maximally 0.5+m2. As a result, mice were presented with more trials on the non-preferred lick spout (i.e. right), gradually and eventually altering the mouse’s preference until it was balanced between spouts. The side-bias correction algorithm was active in pre-training stage 2 and during the first 5 to 7 training sessions of the visual discrimination stage.

### Head-fixed visual two-choice operant conditioning

Two choice (lick left/lick right) operant conditioning featured visual stimuli consisting of sinusoidal gratings, drifting at 1.5 cycles/s. For each mouse, one stimulus was assigned to indicate ‘lick left’ and another to indicate ‘lick right’. These two stimuli were chosen such that each had one of two orientations that differed by 90 degrees, and each had either a low or a high spatial frequency (0.04 or 0.1221 cycles/degree). Selection of stimulus orientation and spatial frequency was counterbalanced across animals. Full contrast stimuli were presented in a 37 degree diameter circular area, centered at 10 degrees elevation and 0 degrees azimuth and blended within an annulus of 4 degrees width into an equiluminant grey background (total stimulus diameter including blended surround was 45 degrees).

Visual discrimination training followed the same basic trial structure as described above (Fig 1E), with the main addition that now a visual stimulus was presented for 2.25 s. The response window (3.0 s duration) started 1.0 s after stimulus onset. During the response window, a lick on the correct spout triggered reward delivery, while a lick on the incorrect spout caused a time-out. On rewarded/correct trials, stimulus presentation was continued for the full 2.25 s. On incorrect trials, the stimulus was replaced by a narrow horizontal black bar spanning the width of the display, presented for the duration of the time-out (2.5 s). Stimulus presentation or time-out was followed by an inter-trial interval of 5.0 s. Licks during the inter-trial interval and in the 1.0 s period between stimulus onset and response window onset (called ‘grace period’) [9] did not change the trial flow. In order to facilitate exploration and motivation, time-outs were not implemented in the first three training sessions. Therefore, in these initial sessions, an incorrect lick did not abort the response window and the mouse could still obtain a reward by subsequently licking on the correct spout.

### Data analysis

Experimental and behavioral parameters such as the timing of licks, timing of drops, running speed, stimulus onset and other triggers were extracted from the passive data-recorder at 2 ms temporal resolution (LabVIEW, National Instruments) and analyzed using custom-written Matlab (Mathworks) and Python routines.

Continuous home cage activity patterns were calculated from the 12 sensors of the DVC system using a custom analysis program (written in Python). Each sensor provided a constant signal (4 Hz), which dropped when a mouse moved near/over it. The variance of the sensor signal was calculated within time bins of 1 minute and subsequently averaged across sensors, resulting in a minute-by-minute indication of average home cage activity per single housed animal. Per mouse/cage, outlying values (>95 percentile) were clipped to the value of the 95^th^ percentile; these outliers often coincided with cage removal from or insertion into the rack. Next, all data points were normalized per mouse/cage (by division) to the 85^th^ percentile of all values recorded during baseline periods (the 7 day period before surgery and the 14 day period before food/water restriction).

Learning performance was calculated as fraction of correct trials per session and evaluated across training sessions. The resulting learning curve was fit with a sigmoidal curve (Eq 3) where x was the average fraction correct trials per session, and parameters y_0_ (minimum of curve), c (maximum of curve relative to y_0_), k (steepness) and x_0_ (time point of maximum steepness) were estimated using least squares fitting.

$$Fitted\;curve=y_{0}+\frac{c}{1+e^{-k(x-x_{0})}}$$ Eq 3

Latency to learning was determined as the number of sessions until an animal exceeded the criterion of 66% correct trials. The behavioral threshold of 66% correct trials was determined based on prior experience. The probability of detecting a single false-positive behavioral threshold crossing across the 23 sessions of the learning curve was 0.001 (assuming 100 trials per session). Maximum learning curve performance was estimated per mouse from the maximum of the individually fitted learning curve. This measure approximates the average level of performance that mice reached after 23 training sessions, independent of the latency to criterion. The total amount of water or soymilk that a mouse consumed during the task was computed from the number of drops that the mouse received. Data are presented as mean ±SD unless mentioned otherwise. Between-group statistical comparisons were carried out using a Mann-Whitney U test.

## Results

### Adaptation to reversed day/night cycle, surgery and recovery

**Fig 2:**
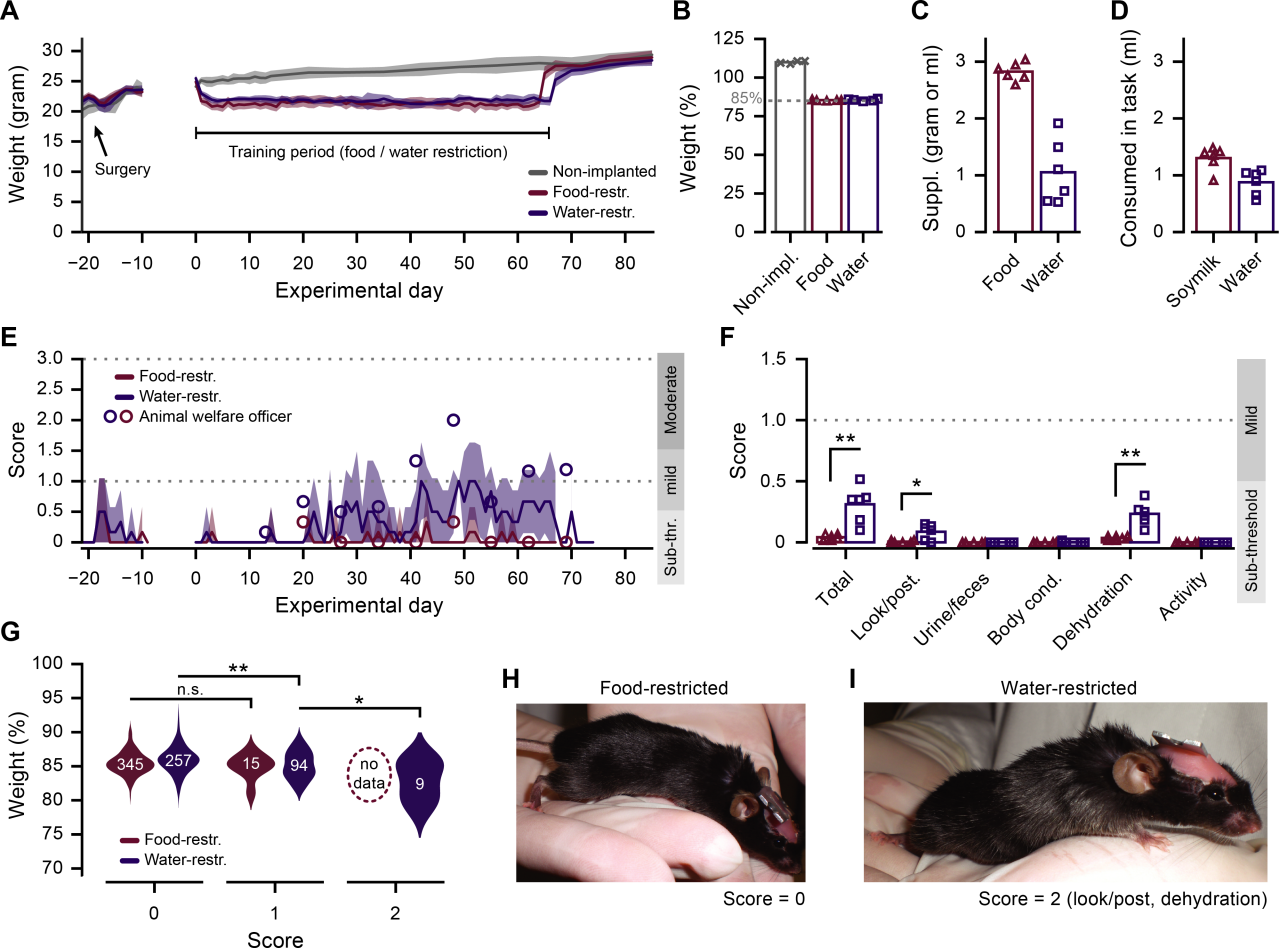
Animal weights and discomfort scores. A. Mean (±SD) daily weight of each experimental group across the entire experiment (gray: non-implanted, n = 4; red: food-restricted, n = 6; blue: water-restricted, n = 6). B. Average weight, in percentage of reference weight, throughout the period of food or water restriction. C. Amount of supplemented food (red) or water (blue) given (average of entire training period). D. Amount of soymilk (red) or water (blue) earned during training (average of entire training period). E. Mean (±SD) daily score of food (red) and water (blue) restricted mice over the entire experiment. Circles indicate scores as judged by the animal welfare officer. F. Daily score averaged across the period of food/water restriction. Total score is the sum across all five individual scores (look/posture, urine/feces, body condition, dehydration signs, activity; MWU test, *p = 0.018, ** p = 0.002). G. Distribution of daily measured weight as a function of the daily determined discomfort score for food- and water-restricted mice (MWU test, *p = 0.016, ** p = 0.002). Numbers in distribution plots indicate n in individual daily measurements. H. Example photo of food-restricted mouse (discomfort score 0, ‘sub-threshold’). I. Example photo of water-restricted mouse (discomfort score, total = 2, ‘moderate’; look/posture = 1; dehydration signs = 1). All panels: grey crosses (non-implanted), red triangles (food-restricted) and blue squares (water-restricted) indicate averages for individual animals.

Four weeks before starting food or water restriction, C57Bl/6j mice were transferred from a local animal breeding facility into individual 24hr/day activity monitoring cages (DVC, see Methods) that were kept in an animal holding room with a reversed day/night cycle. After an adaptation period of 9 to 10 days, the 12 animals with the highest bodyweight were randomly assigned to two experimental groups (food or water restriction, n = 6 each), implanted with a head-bar and subsequently allowed to recover until the start of the experiment. The four remaining mice (having the lowest body weight on the two surgery days) were not implanted and were left kept in their home cage throughout the experiment. While all implanted animals showed a reduction in body weight on the days immediately following surgery, both experimental groups recovered within seven days to a body weight that was comparable to the non-implanted group (Fig 2A).

### Animal wellbeing during food or water restriction

The *ad libitum* reference weight of all mice was taken at 14:00 on day zero, after which food or water restriction was started (see Methods). The body weight of each mouse was maintained at around 85% of the individual *ad libitum* reference in all mice throughout the period of restricted food or water access (Fig 2A and 2B). All animals received a daily individually calibrated supplement of solid food (chow) or water (see Methods; Fig 2C) in addition to any soymilk or water they obtained during handling or training (Fig 2D).

Daily discomfort scores were assessed from the day of the surgery until 10 days after the end of food/water restriction (Fig 2E, solid lines). In addition, the institutes animal welfare officer assessed scores weekly, each Monday at 09:00, during the period of food/water restriction (Fig 2E, circles). The daily assessment of discomfort during the course of the experiment ranged mostly from ‘sub-threshold’ to ‘mild’, but individual scores very occasionally exceeded into the ‘moderate’ range. Signs of discomfort were most often observed in the post-surgery period and from the third week of food/water restriction onwards (Fig 2E).

The average score during the period of food/water restriction remained well below the cut-off for ‘moderate’ discomfort (Fig 2F). However, the food-restricted group had significantly lower total scores compared to the water-restricted group (total: food-restricted, score = 0.04±0.02; water-restricted, score = 0.31±0.13; MWU test, p = 0.002; n = 12 mice). This difference was mostly caused by observations of mild skin turgor (signs of dehydration: food-restricted, score = 0.04±0.01; water-restricted, score = 0.23±0.09; MWU test, p = 0.002; n = 12 mice) and slightly erected, shaggy fur (look/posture: food-restricted, score = 0.01±0.01; water-restricted, score = 0.09±0.06; MWU test, p = 0.018; n = 12 mice). Scores on the other aspects did not exceed zero, except for a single occurrence of a score for the body condition of a water-restricted mouse.

The body weight of water-restricted mice with a total score above zero was on average significantly lower than that of mice with a zero score (score 0: 85.9±1.9%; score 1: 85.2±2.0%; score 2: 82.9±2.8%; MWU test; 0 vs. 1, p = 0.002; 1 vs. 2, p = 0.012; n = 360 scores; Fig 2G). This relation did not hold for food-restricted animals, probably because of the overall very low occurrence of >0 scores in this group (score 0: 85.5±1.6%; score 1: 84.9±1.7%; MWU test; 0 vs. 1, p = 0.30; n = 360 scores). In general, it is important to note that the differences between scores can be quite subtle, as is illustrated in Fig 2H and 2I, depicting a mouse with a score of 0 next to another one that had a total score of 2 (look/posture = 1; signs of dehydration = 1).

### Continuous monitoring of physical activity in the home cage

**Fig 3:**
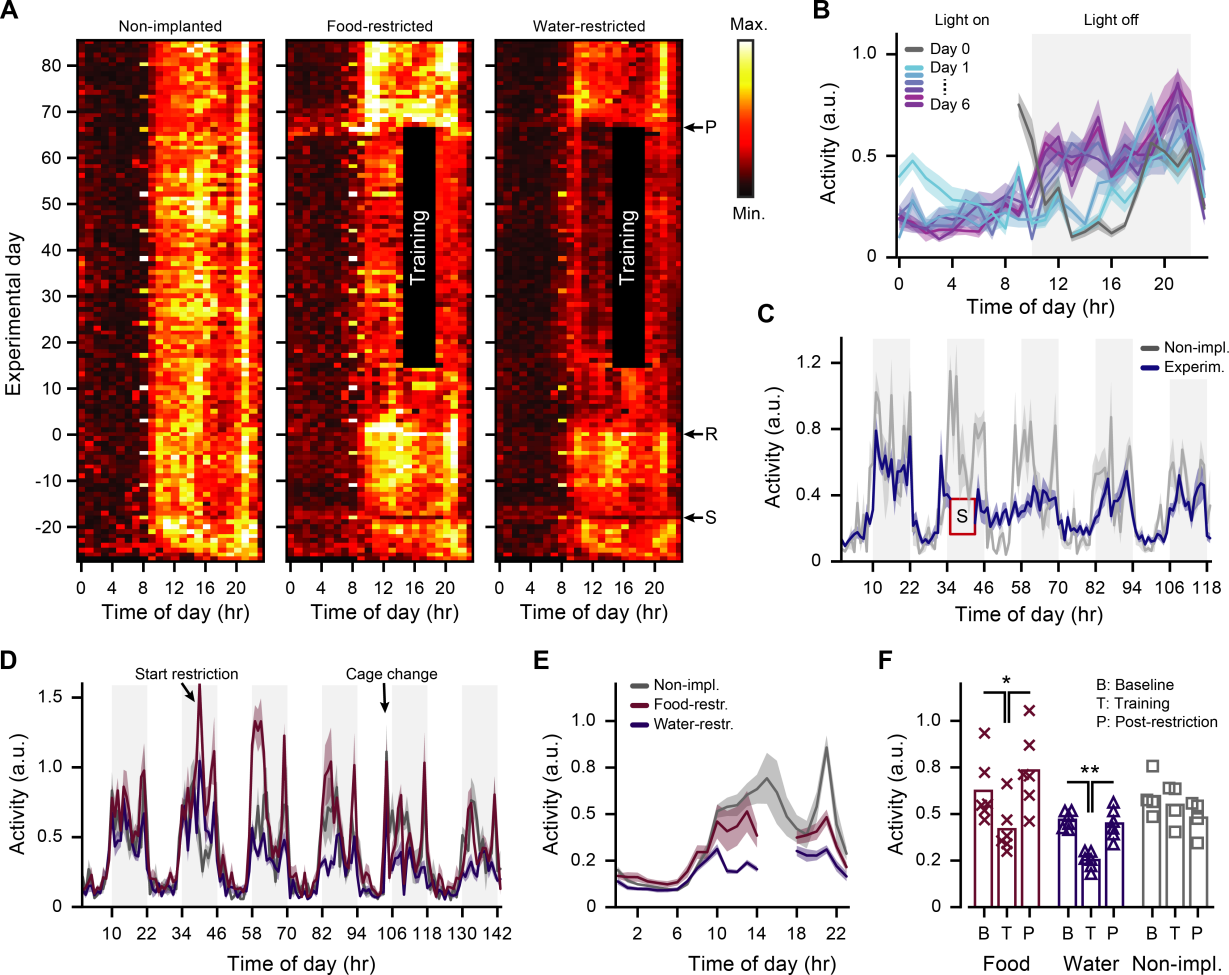
Continuous monitoring of physical activity in the home cage. A. Heat maps depicting baseline-normalized physical activity per hour (x-axis) throughout the days of the experiment (y-axis) as measured in the home cage, averaged across the non-implanted and experimental groups separately. Arrows: ‘S’ indicates the two days on which surgeries were performed; ‘R’ the day on which food or water restriction started; ‘P’ start of the post-training period (note that the food-restricted group received *ad libitum* access to food from two days before this post-training period). Cage changes can be identified as single bright data points, weekly reoccurring at 08:00. B. Hourly averaged (±SEM) home cage activity for the first seven days of adaptation to the reversed day/night cycle. C. Hourly averaged (±SEM) activity centered on the day of head bar implantation (blue, experimental group) or a matched day for animals that did not receive a head bar (gray, non-implanted group). D. Six days of average hourly home cage activity (mean±SEM), starting one day before onset of food or water restriction. E. Average (±SEM) 24hr home cage activity pattern throughout the entire period of training. Data of the experimental groups during the period of training (14:00–18:00) were left out. F. Mean (±SEM) home cage activity in the (active) light-off period (training period excluded). ‘B’: Baseline period, day -14 to 0. ‘T’: Training period, day 1 to 66. ‘P’: Post-restriction period, day 67 to 85. Crosses, triangle and squares indicate data points from individual mice (* MWU test, p<0.02; ** MWU test, p = 0.003).

While the discomfort score featured an instantaneous assessment of physical activity of the mice (activity and behavior), this could not be assessed without disturbing the mice in the first place. In order to measure activity of mice during the entire 24-hour cycle, we recorded the activity of each mouse in its home cage. Individual measurements were normalized to baseline activity as observed before the start of food/water restriction (see Methods; Fig 3A). These continuous readings were sensitive enough to measure the gradual adaptation to the reversed day/night cycle during the first seven days of the experiment (Fig 3B) and alterations to the day/night rhythm during the first two days after head-bar implantation surgery (Fig 3C).

Continuous home-cage activity recordings allowed us to monitor both the acute and long-term effects of restricted access to food or water. During the first few hours after restriction commenced, both experimental groups showed increased activity as compared to the non-implanted (non-restricted) group, which might be explained by the change into a novel cage (Fig 3D). On the following days, water-restricted animals showed a gradual decline in their daily activity, while food-restricted mice initially increased their home cage activity (Fig 3D). This initial increase in activity could indicate food-seeking/digging behaviors, before the animal learns that such efforts go unrewarded.

Across the entire duration of restriction, both food- and water-restricted mice showed reduced activity in the (active) daily light-off period (10:00–22:00, excluding the period during which training was typically done), as compared to their respective baseline levels before restriction had started (food-restricted: baseline = 0.62±0.16; training = 0.42±0.12; MWU test, p = 0.015; n = 6 mice; water-restricted: baseline = 0.47±0.04; training = 0.25±0.04; MWU test, p = 0.003; n = 6 mice; Fig 3E and 3F). This reduction in activity, relative to baseline activity, was not significantly different between food- and water-restricted mice (food-restricted: activity percentage of baseline = 68.0%±14.6%; water-restricted: activity percentage of baseline = 53.6%±5.9%; MWU test, p = 0.0641; n = 12 mice). Finally, in the post-restriction period, during which food and water was available *ad libitum* again, the average daily activity returned to levels that were comparable to the pre-training baseline (food-restricted: post-restriction = 0.73±0.19; water-restricted: post-restriction = 0.45±0.07; Fig 3F). Thus, by using continuous home-cage recordings we observed that food and water restriction induced a reversible reduction of overall activity levels that went undetected using the instantaneous scoring method.

### Operant behavior and task-motivation

**Fig 4:**
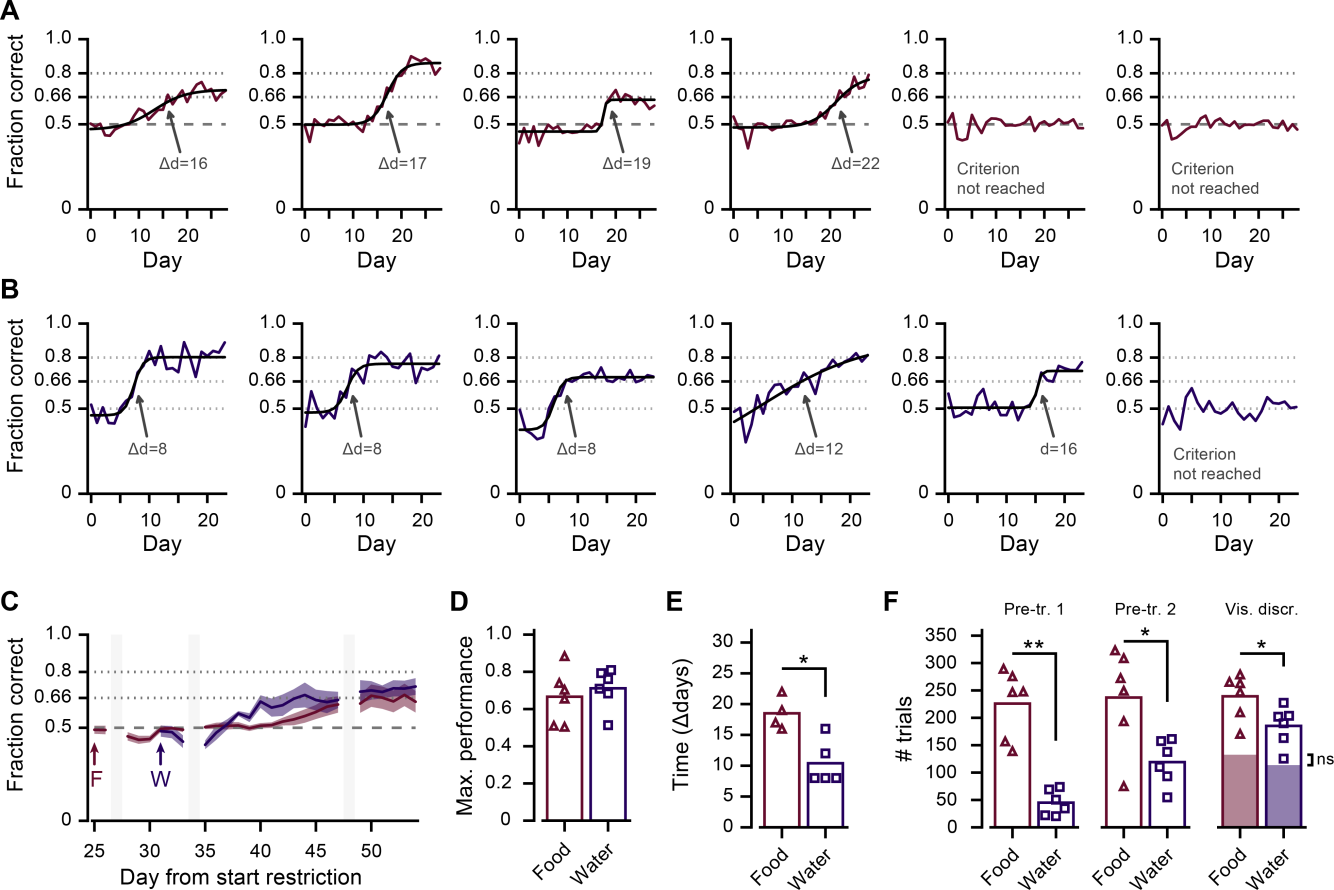
Visual discrimination in a head-fixed two-choice task. A, B. Learning curves of food (A) and water (B) restricted mice. Red and blue lines show the day-to-day performance for each animal, starting at the first day of visual discrimination learning. Black curve is a sigmoidal fit to data from animals that reached criterion (>66% correct). Gray arrows indicate the day on which mice reached criterion. C. Mean (±SEM) learning curve of all food- (red) and water- (blue) restricted mice in the overall experimental timeline. ‘F’ and ‘W’ indicate start days of training food- and water-restricted mice. Gray bars denote days without training. D. Maximum learning curve performance, determined by the sigmoidal fit in the time period during which mice were trained (as shown in A and B). E. Average number of days until criterion (>66% correct) was reached (MWU test, *p = 0.038). F. Average number of trials that mice performed per day in each of the training stages (‘Pre-tr 1’: pre-training stage 1, 1 lick spout; ‘Pre-tr 2’: pre-training stage 2, 2 lick spouts; ‘Vis. Discr.’: visual discrimination task (training stage 3); MWU test, ns: not significant, *p = 0.023, **p = 0.003). The red/blue shaded area in bars of the visual discrimination stage indicates the fraction of rewarded (correct) trials.

To compare how well the method of food and water restriction motivated mice to work for reward in a behavioral paradigm, we compared the total number of completed trials that mice did in the pre-training stages (where every finished trial resulted in delivery of 8 μl soymilk or water). In pre-training stage 1, and (to a lesser extent) in pre-training stage 2, food-restricted mice executed significantly more trials compared to water-restricted animals (pre-training 1, # trials, food-restricted: 226±57; water-restricted: 45±21; MWU test, p = 0.003; pre-training 2, # trials, food-restricted: 237±84; water-restricted: 119±38; MWU test, p = 0.023; n = 12 mice; Fig 4F). As a direct consequence of this difference in total trial number, water-restricted mice required more pre-training stage 1 and pre-training stage 2 sessions to reach criterion compared to food-restricted animals. While we have no clear explanation for this, we noted that in subsequent experiments in our laboratory using water-restriction mice needed fewer pre-training sessions (three to five pre-training stage 1 and two to four pre-training stage 2 sessions). This indicates that potentially subtle changes in procedures, e.g. the experimenters becoming more experienced with the sub-millimeter positioning of the lick spouts, or only a single experimenter carrying out mouse handling and training, can reduce the number of pre-training sessions that water-restricted mice need.

In the final training stage (visual discrimination) the difference between the two groups was less pronounced, even-though food-restricted mice still performed a significantly larger number of trials compared to water-restricted mice (total # trials, food-restricted: 239±38; water-restricted: 185±33; MWU test, p = 0.023; Fig 4F). However, when only considering the trials in which mice made a correct choice, and thus received the soymilk or water reward, food- and water-restricted animals performed approximately equal numbers (Rewarded/correct # trials, Food-restricted: 132±26; Water-restricted: 114±28; MWU test, p = 0.149; n = 12 mice; Fig 4F).

The fact that water-restricted mice performed a lower number of trials throughout all training stages could indicate an overall lower motivation to work for water reward. Alternatively, water-restricted mice might satiate faster from water compared to how fast food-restricted mice satiate from soymilk, and therefore completed fewer trials. Anticipatory licking is a reward-oriented behavior that is related to task-motivation and that can be computed for individual trials [30]. By counting anticipatory licks in a 1 s period from stimulus onset until the response window started (Fig 1E) and averaging over only trials in which animals produced an operant response, we approximated task-motivation independent of satiation. This measure showed that during the final training sessions of the experiment, both food- and water-restricted mice made roughly 3 anticipatory licks per single trial (anticipatory # licks, last 10 sessions, food-restricted: 2.79±1.21; water-restricted: 2.93±0.42; MWU test, p = 0.189; n = 12 mice). However, during the first training sessions of the visual discrimination stage (stage 3), food-restricted mice systematically made fewer anticipatory licks compared to water-restricted mice (anticipatory # licks, first 10 sessions, food-restricted: 1.73±0.90; water-restricted: 3.30±0.91; MWU test, p = 0.015; n = 12 mice). This argues that the lower number of trials that water-restricted mice performed in each training stage did not reflect reduced motivation to lick for reward. Quite the opposite: the results rather suggest that water-restricted mice were even more motivated, but probably satiated faster compared to food-restricted animals.

To confirm that satiation is an important factor in task-motivation, we tested whether there was a correlation between the relative weight of a mouse and its behavioral drive. We found that the daily measurement of relative body weight significantly predicted the number of trials that a mouse would perform in the training session of the same day (Correlation of percentage body weight and total number of trials, z-scored per mouse; food-restricted: r = -0.22, p = 0.005; water-restricted: r = -0.47, p = 3.2∙10^−10^). In summary, food and water restriction can both be used to motivate animals in an operant task, but the total number of trials that mice perform depends on the restriction paradigm.

### Operant learning and performance

To test for differences in operant learning, mice were trained to discriminate visual patterns in the two-choice head-fixed lick left/lick right task. Out of twelve mice, four food-restricted and five water-restricted mice reached the performance criterion of 66% correct trials on a given day (Fig 4A and 4B). For all mice, maximum learning curve performance was estimated from the fitted learning curve on the last day of training and did not differ between groups (Maximum of fitted learning curve, food-restricted: 0.67±0.13; water-restricted: 0.71±0.10; MWU test, p = 0.189; n = 12 mice; Fig 4C and 4D). However, water-restricted mice reached the criterion of 66% correct trials significantly faster compared to food-restricted mice (food-restricted, Δdays = 18.5±2.3; water-restricted, Δdays = 10.4±3.2; MWU test, p = 0.038; n = 9 mice; Fig 4C and 4E). This difference did not depend on the exact value of the threshold. A similar difference was observed with a higher threshold (70%, as in Guo et al., 2014; food-restricted, Δdays = 21.0±2.9; water-restricted, Δdays = 11.0±3.0; MWU test, p = 0.033; n = 9 mice) as well as with a lower threshold (60%; food-restricted, Δdays = 17.3±1.9; water-restricted, Δdays = 5.8±2.4; MWU test, p = 0.003; n = 10 mice). Also, using an altogether different method of quantifying whether the learning criterion was reached, the number of training sessions to reach the point of maximum steepness of the sigmoid fitted learning curve, we observed that water-restricted mice learned faster (food-restricted, Δdays = 18.0±2.8; water-restricted, Δdays = 7.8±4.8; MWU test, p = 0.046; n = 9 mice).

To investigate whether motivational state or satiation could explain the difference in speed of learning, we tested whether either the average relative weight-loss of a mouse, or the average number of anticipatory licks in 10 pre-learning sessions, predicted the number of sessions needed to reach learning criterion. However, neither variable correlated significantly with learning speed (correlation of percentage body weight and time to reach criterion, z-scored per condition: r = 0.11, p = 0.77; correlation of # of anticipatory licks and time to reach criterion, z-scored per condition: r = -0.54, p = 0.14; n = 9 mice). Additionally, we tested whether day-to-day fluctuations in relative body weight predicted task performance on the corresponding day (in mice that performed above criterion), which also did not correlate significantly in either the food- or water-restricted group (correlation of session-wise percentage body weight and performance, z-scored per mouse; food-restricted: r = -0.19, p = 0.28, n = 34 sessions; water-restricted: r = 0.11, p = 0.43, n = 58 sessions).

Another factor that may influence learning is general locomotor activity such as wheel-running in the home cage [31]. However, while food and water restriction both led to an overall reduction of home cage activity (Fig 3F), we observed that the most active mice actually took the longest to reach criterion (correlation of mean DVC activity and time to reach criterion, z-scored per condition: r = 0.82, p = 0.0069; n = 9 mice). In contrast to home-cage activity, we noted a large difference in the amount of running that the two groups of mice did during the visual discrimination task. Here, the water-restricted group ran about double the distance of the food-restricted group (distance ran per training session, food-restricted: 33±11 m; water-restricted: 66±24 m; MWU test, p = 0.023; n = 12 mice). Still, day-to-day differences in the amount of in-task running did not predict the performance on the visual discrimination task in either group (correlation of distance ran and performance, z-scored per mouse; food-restricted: r = 0.19, p = 0.28, n = 34 training sessions; water-restricted: r = 0.05, p = 0.72, n = 54 training sessions), and neither did the overall amount of in-task running predict the speed of learning (correlation in-task distance ran and time to reach criterion, z-scored per condition: r = -0.16, p = 0.67; n = 9 mice). Thus, parameters associated with motivational-state and physical activity provided a poor prediction of learning speed or task performance and do not likely explain the difference in time to reach learning criterion of food- and water-restricted mice.

## Discussion

This study provides a detailed behavioral protocol for training mice in a fast and reliable way on a head-fixed two-alternative choice visual discrimination task. Our results show that most of the animals that were trained on the protocol learned discriminating visual stimuli within two or three weeks from the start of visual conditioning. An important aim of this study was to utilize the welfare- and behavioral read-outs of our training protocol to contrast two commonly used methods for motivating animals in head-fixed behavioral paradigms, i.e. food restriction with soymilk reward and water restriction with water reward. Using either method, the animals could be motivated to perform the task at or above criterion, without exceeding the ‘mild’ discomfort category, even for prolonged periods. However, we did observe specific differences in welfare assessment and in task performance, such as time to reach criterion and number of performed trials, which should be considered when selecting the restriction method.

### Operant behavior

Throughout the training stages, there was a systematic difference in the number of trials that water- and food-restricted mice performed per session. In pre-training stage 1 and 2, food-restricted mice consumed larger volumes of soymilk than water-restricted mice consumed water. Furthermore, food-restricted animals proceeded faster through the pre-training stages than water-restricted mice. These differences might be explained by water-restricted mice satiating faster than food-restricted mice, since the 8-microliter water reward equaled on average 0.43% of the daily water intake in our experiment (1.93 ml), and the 8-microliter soymilk reward provided only 0.05% of the daily caloric food intake in this study (11.15 kcal). However, in pre-training 1 sessions water-restricted mice performed on average only 50 trials, which is approximately only 25% of their daily water ration. Possibly, water-restricted mice already satiate after 40–50 drops and only through experiencing multiple pre-training sessions learn to obtain more water than they acutely need. On the other hand, Guo et al. [9] observed that water-restricted mice performed more trials when sucrose was added to the water reward. Similarly, in our experiment we noted that water-restricted mice after reaching criterion performed more trials when provided with soymilk reward compared to the usual water reward (data not shown). Therefore, soymilk reward may have had additional motivating or appetitive aspects compared to plain water reward. Possibly, this is related to the nutrients and flavor that soymilk contains. Alternatively, it is conceivable that the smell of reward (soymilk) coming directly from the lick spouts made it easier for food-restricted (soymilk rewarded) mice to learn the initial behavior of licking for reward.

Despite water-restricted mice performing fewer trials per session, they were on average faster in reaching the learning criterion (independent of which exact criterion we used). Throughout the experiment, we aimed for keeping the motivational state of individual animals comparable by maintaining the relative weight of each animal at 85% of the *ad libitum* measured reference value. In addition, we excluded that the difference in learning speed correlated with parameters reflecting task-motivation in this study. One remaining explanation could be that, as described above, the 8-microliter water drop might have been subjectively perceived as a larger reward compared to a soymilk drop of the same volume, thus providing a greater learning incentive for water-restricted mice. Moreover, there are fundamental differences in the neural circuits that mediate hunger and thirst [22], asserting different effects on motivation and learning that could provide a stronger incentive for learning in one group compared to the other. Importantly, the speed of learning, maximum learning curve performance and success rate achieved using either restriction method in this study was similar to previously reported head-fixed operant conditioning paradigms, e.g. [6,9].

A final in-task difference between food- and water-restricted animals was the distance they ran on the Styrofoam ball during the period of behavioral training, with food-restricted mice running significantly less than water-restricted mice. While speculative, one possible explanation is that water-restricted mice are in a higher anticipation state during the task, as they receive relatively more of their daily water amount within-task compared to the relative caloric amount that food-restricted mice receive during the task, leading to hyperactive behavior [32]. Another explanation is that water-restricted mice spend more time running because they performed fewer trials in each training session and therefore had more time in which they were not task-engaged.

### Welfare assessment

We aimed to facilitate the objective categorical distinction between methods of food and water restriction by maintaining mice in both conditions close to a body weight of 85% of their baseline reference. Still, there remained a limited amount of day-to-day and mouse-to-mouse variation of relative body weight within the protocol. These fluctuations correlated with the number of trials that mice performed in the behavioral task, showing that relative weight loss is a key factor in task-motivation. In case of water-restricted mice, relative weight loss also corresponded to the daily discomfort score (Fig 2G). One reason the absence of a correlation between relative weight loss and discomfort scores in food-restricted mice could be that this group had mostly discomfort scores of zero. Alternatively, it is conceivable that scores directly attributable to dehydration, such as skin tenting and fur appearance, were more sensitive or better observable compared to scores related to reduced food intake, such as the body condition score.

Although we only once observed reduced activity using the instantaneous scoring method, both food- and water-restricted mice showed less home-cage activity in the continuous home-cage recordings compared to their own baseline measurement. In food-restricted mice, the reduction in overall home-cage activity could reflect reduced food-seeking behavior. In water-restricted mice, the reduced home-cage activity might (in part) reflect a decrease in grooming behavior, which could be a factor contributing to their higher score on the parameter ‘look/posture’ in the overall health assessment. Indeed, water loss in the form of saliva used for grooming can account for up to one third of water loss in rodents that are not water-restricted [33]. It is possible that water-restricted mice conserve water by reducing the amount of grooming, leading to overall poorer fur appearance.

It should be noted that we did not assay the effect of food or water restriction on the mouse’s physiology and neuronal circuitry, neither did we measure the effect of water restriction on food-intake behavior. In addition, the five scoring parameters may have differed in their sensitivity for detecting food- or water restriction associated discomfort. Therefore, we do not aim to draw conclusions from the differences in scores between restriction regimens observed in this study, but rather advise considering these results in the context of literature on food and water restriction procedures, e.g. [20,23,34,35]. Furthermore, the choice for setting a threshold at 85% of pre-restriction body weight is rather arbitrary. Other studies use different thresholds, either above or below 85%, and occasionally take into account the gradual increase in weight that would be observed in non-restricted mice, e.g. [6,36–38]. Still, these methods do not consider that there may be individual variation in how mice adapt to chronic water restriction [35,39]. Therefore, in our opinion, the best method would be to set the threshold for continuation of an experiment entailing food or water restriction using the measure of discomfort directly, as for instance described in Guo et al. [9], and monitor the relative weight of the animals as an indication, but not as threshold.

### Practical considerations

In the last two decades, the mouse has gained increasing attention in neuroscience as a versatile research model that can be adopted for studying sensory processing, learning and memory, decision making and motor behavior under both healthy and diseased conditions. Our behavioral protocol and conditioning task for training head-fixed mice can be readily combined with *in vivo* recording techniques such as intracellular patch clamp recordings [40], two-photon microscopy [13], but also with newly developed techniques for single cell control of neuronal activity patterns [41]. The two-choice lick left / lick right task can be easily adapted to include other sensory modalities, or expanded for the study of higher cognitive functions, making it a useful tool for studying mouse behavior in general. In addition, the in-task differences we observed between food- and water-restricted animals can be exploited in order to suit the specific behavioral requirements. Finally, we showed that the use of a continuous home-cage monitoring system allows expanding the quantification of animal wellbeing to include an objective measure of overall activity, which allows observing light-cycle adaptation, post-surgery recovery and effects of food and water restriction without disturbing the animals. Behavioral paradigms will likely always require precise fine-tuning of a large, mostly un-documented parameter space. The methods and procedures described in this study are intended to guide this process to smoother convergence while improving animal wellbeing.

## Supporting information

S1 File Head bar holder design. These files contain the designs of the head bar and of the components necessary for building the head bar holder. The files were produced in SolidWorks (Dassault Systèmes) and can also be viewed using the free program eDrawings (http://www.edrawingsviewer.com).
(ZIP)

S2 File Lick spout holder design. This file contains the design of the 3D printable lick spout holder. It was produced and can be opened using the online service TinkerCat (https://www.tinkercad.com). The file can also be opened with the free program eDrawings (http://www.edrawingsviewer.com) as well as with most software delivered with 3D printers.
(STL)
